# The need of health policy perspective to protect Healthcare Workers during COVID-19 pandemic. A GRADE rapid review on the N95 respirators effectiveness

**DOI:** 10.1371/journal.pone.0234025

**Published:** 2020-06-03

**Authors:** Primiano Iannone, Greta Castellini, Daniela Coclite, Antonello Napoletano, Alice Josephine Fauci, Laura Iacorossi, Daniela D’Angelo, Cristina Renzi, Giuseppe La Torre, Claudio M. Mastroianni, Silvia Gianola

**Affiliations:** 1 Centro Eccellenza Clinica, Qualità e Sicurezza delle Cure, Istituto Superiore di Sanità, Rome, Italy; 2 Unit of Clinical Epidemiology, IRCCS Istituto Ortopedico Galeazzi, Milan, Italy; 3 Institute of Epidemiology & Health Care, University College London-UCL, London, United Kingdom; 4 Department of Public Health and Infectious Diseases, Sapienza University of Rome, Rome, Italy; Universita degli Studi di Ferrara, ITALY

## Abstract

Protecting Health Care Workers (HCWs) during routine care of suspected or confirmed COVID-19 patients is of paramount importance to halt the SARS-CoV-2 (Severe Acute Respiratory Syndrome-Coronavirus-2) pandemic. The WHO, ECDC and CDC have issued conflicting guidelines on the use of respiratory filters (N95) by HCWs. We searched PubMed, Embase and The Cochrane Library from the inception to March 21, 2020 to identify randomized controlled trials (RCTs) comparing N95 respirators versus surgical masks for prevention of COVID-19 or any other respiratory infection among HCWs. The grading of recommendations, assessment, development, and evaluation (GRADE) was used to evaluate the quality of evidence. Four RCTs involving 8736 HCWs were included. We did not find any trial specifically on prevention of COVID-19. However, wearing N95 respirators can prevent 73 more (95% CI 46–91) clinical respiratory infections per 1000 HCWs compared to surgical masks (2 RCTs; 2594 patients; low quality of evidence). A protective effect of N95 respirators in laboratory-confirmed bacterial colonization (RR = 0.41; 95%CI 0.28–0.61) was also found. A trend in favour of N95 respirators was observed in preventing laboratory-confirmed respiratory viral infections, laboratory-confirmed respiratory infection, and influenza like illness. We found no direct high quality evidence on whether N95 respirators are better than surgical masks for HCWs protection from SARS-CoV-2. However, low quality evidence suggests that N95 respirators protect HCWs from clinical respiratory infections. This finding should be contemplated to decide the best strategy to support the resilience of healthcare systems facing the potentially catastrophic SARS-CoV-2 pandemic.

## Introduction

The Severe Acute Respiratory Syndrome-Coronavirus-2 (SARS-CoV-2) outbreak emerged in China in December 2019 and it was recognised as a pandemic by the World Health Organization (WHO) on 11 March [1]. As of 3 May 2020, a total of 3,349,786 cases and 238, 628 deaths have been reported worldwide [2]. Nosocomial spread and infection of healthcare workers (HCWs) are a major concern. In Italy HCWs are paying a heavy price in addition to their professional and humanitarian efforts, with 21,338 cases (more than 10,4% of total Italian cases [3]) and 154 deaths [4] among physicians. Protecting HCWs from SARS-CoV-2 is therefore of great importance for individual HCW and for their role in fighting this devastating pandemic effectively. Claims of insufficient protection of HCWs by personal protective equipment, in particular with regards to the use of surgical masks, have fuelled the scientific and social media debate in several countries. While both surgical masks and N95 respirators are worn by HCW for self protection, they have different intended uses [5]. Surgical masks do not prevent inhalation of small airborne particles and fit the face loosely while N95 respirators are able to do so by fitting tightly to the wearer’s face and fulfil strict filtration requirements. In fact, except for aerosol generating procedures requiring higher level of respiratory protection with filtering respirators (ie. N95 respirators), WHO considers surgical masks adequate for the routine care of coronavirus disease 2019 (COVID-19) patients [6]. Instead, the Centers for Disease Control (CDC) and the European Center for Disease Control guidelines (ECDC) have a more cautious approach, acknowledging that the exact role of airborne (aerosol) route in the transmission of SARS-CoV-2 is still largely unknown [7, 8]. The direct evidence supporting the WHO guidelines is based on very few case reports on the absence of SARS-CoV-2 in air samples taken in highly protected environments where a rapid dilution of aerosols occurs, the absence of infection of HCWs exposed for a limited time or limited viral loads, or on modelling of epidemiologic patterns of transmission [9–12]. In contrast, the airborne (aerosol) opportunistic route of transmission has been documented for SARS and MERS caused by closely related coronaviruses responsible of severe nosocomial infections among HCWs. Aerosol filtering respirators were consequently recommended for SARS during 2002–03 outbreak [13]. It is worth remembering that Canadian Health authorities modified their earlier recommendations in favour of a more strict respiratory protection after the deaths of several HCWs [14]. The presence of SARS-CoV-2 in aerosols has been documented in experimental [15] and real life conditions in crowded, poorly ventilated hospital areas unrelated to aerosol generating procedures [16]. Also, spontaneous cough generates aerosols, not only droplets [17, 18] and COVID-19 patients may infect HCWs in this way, especially if they are unable to wear facemasks due to hypoxia and need of oxygen therapy. Moreover, none of the above mentioned guidelines adopted the suggested Grading of Recommendations Assessment, Development and Evaluation (GRADE) approach for directing public health policy decisions and they did not explicitly consider the potentially catastrophic consequences of deferring the recommendation of N95 respirators for HCWs while awaiting more robust evidence.

There are already some systematic reviews addressing the role of N95 respirators in protecting HCWs, offering however a debatable interpretation of the estimates of the effect (ie. reviews’ analyses did not take into account the clustered design of RCTs) [19–22]. We therefore undertook a systematic review with a different perspective and methodology, given the exceptional disease burden expected from this pandemic [23], the central role of protecting HCWs and the need of a careful definition of the outcomes, which are critical for unbiased public health policy decisions [24]. Indeed strengthening the preparedness and resiliency of health care systems to this pandemic crisis occurs not only avoiding SARS-CoV-2 infection but also preventing any HCW respiratory infection causing absenteeism from work. We therefore conducted a systematic review aimed at assessing the efficacy of N95 respirators versus surgical masks for the prevention of respiratory tract infections transmission among HCWs. The evidence from the review can then be used for the development of an appropriate GRADE framework for public health policy guidelines.

## Methods

We conducted this systematic review following the preferred reporting items for systematic reviews and meta-analyses statement (PRISMA) [25] and the Cochrane Handbook for Systematic Reviews of Interventions [26].

### Inclusion and exclusion criteria

#### Types of studies

Randomized controlled trials (RCTs) run in healthcare settings were considered eligible. Randomization was allowed both at individual and cluster level.

#### Population

HWCs exposed to SARS-CoV-2 or any other respiratory infection. *Subgroups*: in-patient versus out-patient hospital setting.

#### Types of interventions

N95 respirators versus surgical masks. An N95 respirator is a particulate-filtering facepiece respirator that meets the U.S. National Institute for Occupational Safety and Health (NIOSH) N95 classification of air filtration, meaning that it filters at least 95% of airborne particles. N95 respirators are considered functionally equivalent to certain respirators regulated under non-U.S. jurisdictions, such as FFP2 respirators of the European Union and KN95 respirators of China. Whereas, the term “surgical mask” was considered equivalent to medical masks (defined surgical, procedural, isolation, laser, fluid resistant or face masks) that meet bacterial and particle filtration efficiency standards required by the US Food and Drug Administration (ASTM standard F2100–11) but are not certifiable as N95 respirators. [22]

#### Types of outcomes and assessment measures

*As suggested by the Cochrane Handbook for Systematic Reviews of Interventions [26],* we identified a priori the following outcomes:

- *Primary outcomes*:
  (i) SARS-CoV-2 infection; (ii) Clinical respiratory illness (CRI).
- *Secondary outcomes*:
  (iii) Influenza like illness (ILI); (iv) Laboratory-confirmed respiratory viral infection; (v) Laboratory-confirmed bacterial colonization; (vi) Laboratory-confirmed respiratory infection; (vii) Laboratory-confirmed influenza; (viii) Discomfort of wearing respiratory protections.

Outcome definitions are reported in S1 Appendix. Outcomes and assessment measurements.

### Search strategy

We searched PubMed, EMBASE, and The Cochrane Library databases from inception to March 21, 2020, to identify published randomized controlled trials (RCTs) on evaluating the use of masks for preventing epidemic influenza. Relevant reviews were consulted for additional studies to consider. The full search strategy is reported above. The full search strategy is reported in S1 Appendix. Search strategy.

### Study selection and data extraction

Two reviewers independently screened the articles based on the titles, abstracts and full texts. Then, two reviewers independently extracted the following data from the included studies: first author, publication year, country, type of influenza detected, season of interest, details of study population and intervention, study design, sample size, settings, and outcome findings. All disagreements were resolved by discussion.

### Risk of bias assessment

Two reviewers independently assessed the risk of bias (RoB) of the selected RCTs using the Cochrane Risk of Bias tool. Also, in cluster-RCTs specific risk of bias were considered. Further details are reported in S1 Appendix. Risk of bias assessment.

### Data analysis and synthesis of results

We pooled data from studies with similar interventions and outcomes (for the intention-to-treat analysis) to calculate relative risk (RR) and the corresponding 95% confidence intervals (CIs). For cluster RCTs, we applied the specific method described in the Cochrane Handbook [27] to account for the clustering and obtain an adjusted CIs. When the cluster RCT did not considered the clustering in the analysis, we multiplied the standard error of the effect estimate (from the analysis ignoring the clustering) by the square root of the design effect: both the number of participants and the number experiencing the event were divided by the same design effect [27]. We then calculated the pooled estimates by using both the fixed-effects and the DerSimonian and Laird random-effects model [28, 29]. In the absence of heterogeneity, the fixed-effects and the random-effects models provide similar results, whereas the random-effects model is considered more appropriate when heterogeneity is found. Heterogeneity between study-specific estimates was tested with the Cochran Q test [30] and measured with the I^2^ statistic [31]. We performed a subgroup analysis based on the settings, ie. in-patients vs out-patients. Publication bias was evaluated with funnel plot if a sufficient number of studies was present. A probability level <0.05 was considered statistically significant, except for heterogeneity, whose level of statistical significance was set at p < 0.10. All statistical analyses were performed using Review Manager (RevMan) version 5.3 [32].

Given the emergency context of this systematic review and the evidence available insofar, we used the results of our meta-analysis to quantify as precisely as possible what value an immediate adoption of N95 respirators for HCWs managing COVID-19 patients could have compared to the added benefit derived from further research. We therefore used the Claxton model [33] to obtain the best estimate of immediate implementation of the N95 respirators through the point estimate of the RR of the corresponding meta-analysis, while the value of deferring the N95 implementation until further research would be available was measured through the upper 90% CI with a time horizon limited to an infectious outbreak. We adopted the worst-case scenario for public health decisions: the least likely benefit which is represented by the lower limit of a confidence interval placed around the treatment effect point estimate [34]. In fact CIs with different levels of confidence can demonstrate that there is differential evidence for different degrees of benefit or harm. For example, it might be possible to report the same analysis results (i) with 95% confidence that the intervention does not cause harm; (ii) with 90% confidence that it has some effect; and (iii) with 80% confidence that it has a patient-important benefit. These elements may suggest both usefulness of the intervention and the need for additional research [35]. See S1 Appendix. Data Analysis and Synthesis of Results.

### Quality of the evidence-GRADE approach

We evaluated the overall quality of the evidence for primary and secondary outcomes using the GRADE approach [32]. Adjusted estimates were considered for judging the quality of the evidence. For primary outcomes, absolute effects were calculated at 95% CI and 90% CI [36]. A ‘summary of findings’ including the quality of the evidence, reasons for limitation and main findings were displayed in table. See S1 Appendix. Quality of the evidence-GRADE approach.

## Results

### Study selection

A total of 390 records resulted from the search on the electronic databases. Overall, we included four RCTs from five publications, of which one was an individual participants randomized trial [37] and three were cluster randomized trials [5, 38, 39]. One publication included additional outcomes related to one cluster RCTs [40].

**Fig 1:**
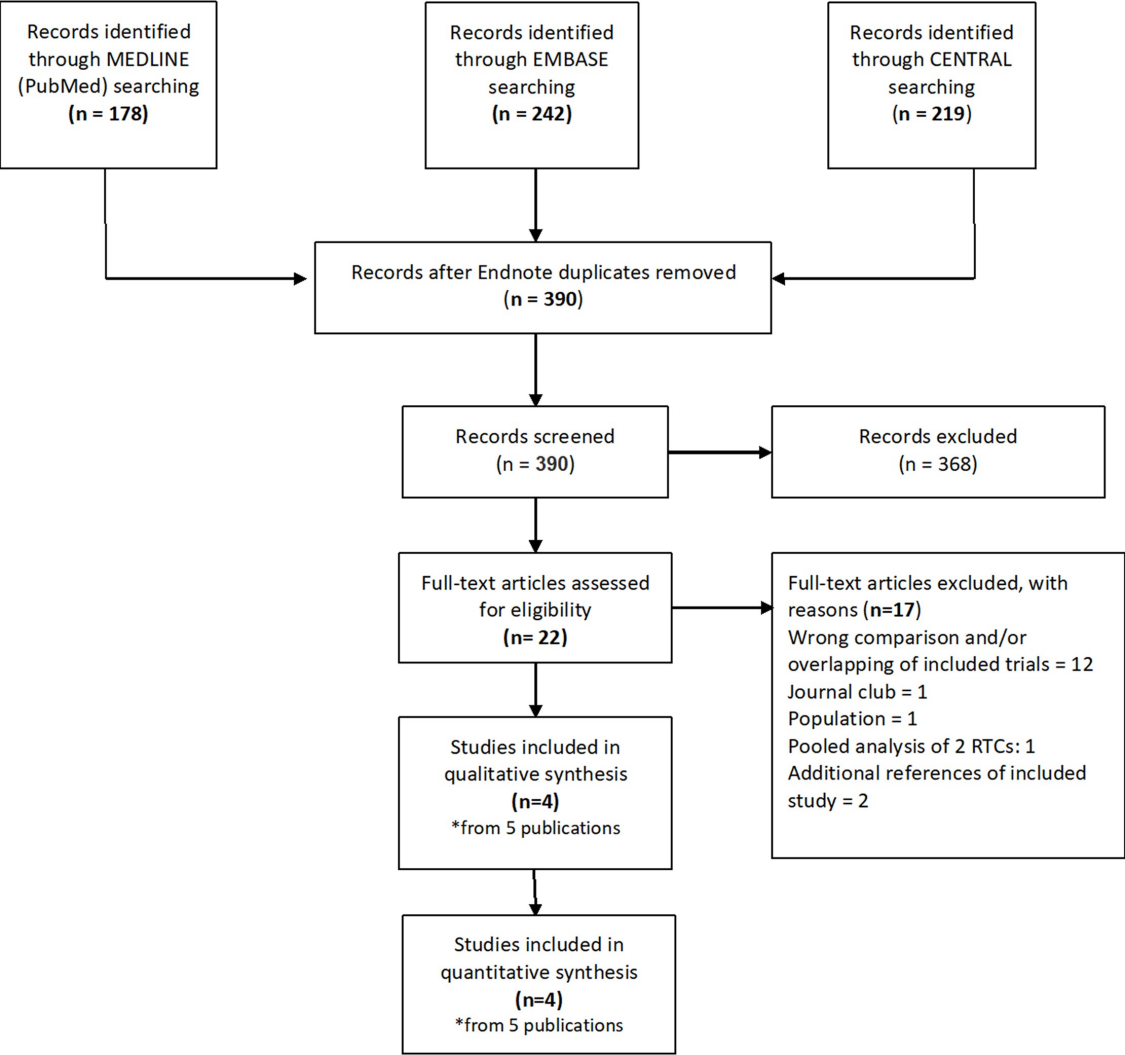
Flow diagram of study selection process.

Fig 1 shows the flow diagram of the study selection process. The list of excluded studies in reported in S1 Appendix. (S1 Appendix. List of excluded studies).

### Description of the included studies

Overall, 8736 participants were considered, with the number of participants for each trial ranging from 446 to 5180. Three cluster randomized studies were performed in an inpatient [5, 38, 39] and one in an outpatient [41] setting.

Table 1. General characteristics of included RCTs.

**Table 1:** General characteristics of included studies.

*Study*	*Study design*	*Setting*	*Participants*	*Influenza–detected and Season*	*Intervention*	*Outcomes*	*Follow up*
**Loeb 2009**	RCT–Non inferiority study	*Hospital* 8 hospitals in Ontario, Canada. enrolled from a total of 22 units, which included 9 acute medical units, 7 emergency departments, and 6 pediatric units.	446 nurses;	2008–2009 influenza SeasonDetection of: influenza A virus subtypes H1 (seasonal), H3, and H5. Parainfluenza virus types 1, 2, 3, and 4; respiratory syncytial virus types A and B; adenovirus; metapneumovirus; rhinovirus-enterovirus; and coronaviruses OC43, 229E, SARS, NL63, and HKU1.	Intervention: N95 respirator Control: surgical mask	Laboratory-confirmed Influenza; laboratory-confirmed respiratory viral infection; influenza-like illness.	5 week follow -up
**MacIntyre et al 2011/2014**	Cluster RCT (by hospital)	*Hospital* 15 hospitals in Beijing, China: emergency departments and respiratory wards.	1441 nurses, doctors and ward clerks	Winter season December 2008 to January 2009. Detection of: adenoviruses, human metapneumovirus, coronavirus 229E ⁄ NL63, parainfluenza viruses 1, 2 or 3, influenza viruses A or B, respiratory syncytial virus A or B, rhinovirus A⁄ B and coronavirus OC43 ⁄ HKU1.	Intervention 1: fit-tested N95 respirator Intervention 2: nonfit-tested N95 respirator Control: surgical mask	Clinical respiratory infection (CRI); laboratory-confirmed influenza; Laboratory-confirmed respiratory viral infection; laboratory-confirmed bacterial colonization; laboratory-confirmed respiratory infection; influenza-like illness.	5 week follow up
**MacIntyre et al 2013**	Cluster RCT (by ward)	*Hospital* 19 hospitals in Beijing, China: emergency departments and respiratory wards	1669 nurses, doctors and ward clerks	December 28, 2009 to February 7, 2010 (winter season). Detection of: adenoviruses; human metapneumovirus; coronaviruses 229E/NL63 and OC43/HKU1; parainfluenza viruses 1, 2, and 3; influenza viruses A and B; respiratory syncytial viruses A and B; or rhinoviruses A/B.	Intervention 1: continual use, fit-tested N95 respirator Intervention 2: targeted use, fit-tested N95 respirator Control: surgical mask	Clinical respiratory infection (CRI); laboratory-confirmed influenza; Laboratory-confirmed respiratory viral infection; laboratory-confirmed bacterial colonization; influenza-like illness.	4 week follow up
**Radonovich et al 2019**	Cluster RCT (by participating sites)	*Hospital out-patient* USA 7 health systems—Outpatient settings serving adult and pediatric patients with a high prevalence of acute respiratory illness (primary care facilities, dental clinics, adult and pediatric clinics, dialysis units, urgent care facilities and emergency departments, and emergency transport services)	5180 nurses/nursing trainees, clinical care support staff, administrative/clerical staff, physicians/advanced practitioners/physician trainees, registrations/clerical receptions, social workers/pastoral cares and environmental service workers/housekeepers.	September 2011 and May 2015, with final follow-up on June 28, 2016. syncytial virus, metapneumovirus, parainfluenza virus, rhinovirus-enterovirus, coronavirus, coxsackie/echovirus	Intervention: fit-tested N95 respirator Control: medical mask	Laboratory-confirmed Influenza; Laboratory-confirmed respiratory infection; influenza-like illness.	12 week follow up

### Risk of bias

In the S1 Appendix (S1 Appendix. Results of Risk of bias assessment) we show the risk of bias of included studies: Loeb et al. 2009 [37] was judged at low risk of bias, the remaining [5, 38–40] were assessed for additional bias related to clustering of which two out of three cluster RCTs were assessed as high risk of bias for imbalance at baseline. Overall, all trials have unclear allocation.

### Primary outcomes

**Fig 2:**
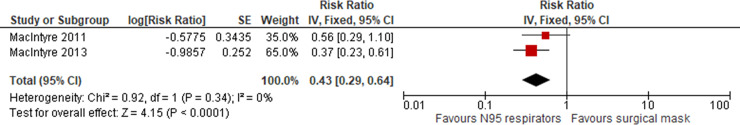
Forest plot of Clinical Respiratory Illness (CRI)–random effect model meta-analysis with 95% CI.

**Fig 3:**
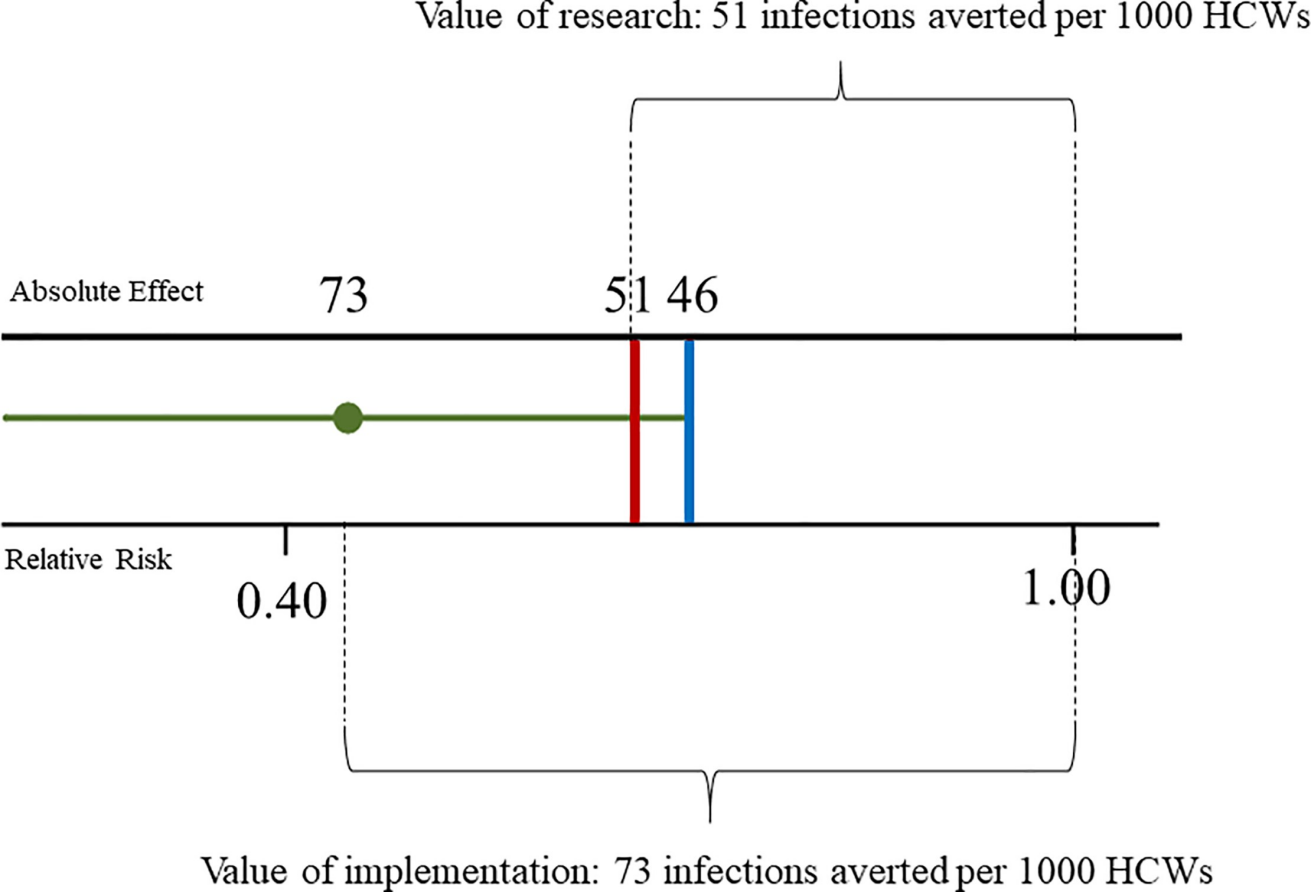
Trade-offs between implementation and deferral of the intervention about Clinical Respiratory Illness (CRI). The green line represents the 95% CI of the overall effect of N95 respirators respect to surgical masks for CRI. The blue line is the upper limit of 95% CI, RR 0.43 [0.29–0.64] anticipated absolute effect: 73 [91 46], the red line is the upper limit of 90% CI, RR 0.43 [0.31–0.60] anticipated absolute effect: 73 [88 51]. Value of implementation is therefore of 73 infections averted x 1000 HCW, value of further research is of 51 infections averted x 1000 HCW.

No RCTs addressing the prevention of SARS-CoV-2 infection among HCWs was found. For CRI, we included two cluster RCTs with 2594 HCWs from in-patient hospital setting [38, 39]. Adjusting data for clustering, using N95 respirators reduced meaningfully the risk of developing CRI respect to surgical masks (2 RCTs, RR 0.43, 95% CI 0.29, 0.64; I^2^ = 0%) (Fig 2), with low quality of evidence and an absolute effect of preventing 73 more (95% CI from 91 more to 46 more) infections per 1000 HCWs wearing N95 respirators (Table 2). According to the Claxton model [33], in the worst case scenario the added benefit of more research in reducing uncertainty would be of reducing to 51 infections (upper 90% CI limit) prevented per 1000 HCWs wearing N95 respirators compared to surgical masks) (Fig 3).

**Table 2:** GRADE summary of findings table.

Outcomes*	№ of participants* (studies) Follow up	Certainty of the evidence (GRADE)	Relative effect (95% CI)	Anticipated absolute effects**	
				Risk with surgical masks Adjusted	Risk difference with N95 respirators
Clinical respiratory illness	1420 (2 RCTs)	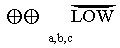	**RR 0.43** (0.29 to 0.64)	128 per 1.000	**73 fewer per 1.000** (91 fewer to 46 fewer)
Influenza like illness	3937 (4 RCTs)	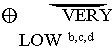	**RR 0.72** (0.38 to 1.37)	42 per 1.000	**12 fewer per 1.000** (26 fewer to 16 more)
Laboratory-confirmed respiratory viral infections	1866 (3 RCTs)	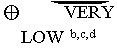	**RR 0.84** (0.52 to 1.34)	46 per 1.000	**7 fewer per 1.000** (22 fewer to 16 more)
Laboratory-confirmed bacterial colonization	1420 (2 RCTs)	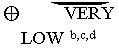	**RR 0.41** (0.28 to 0.61)	145 per 1.000	**86 fewer per 1.000** (104 fewer to 57 fewer)
Laboratory-confirmed respiratory infection	2792 (2 RCTs)	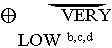	**RR 0.73** (0.40 to 1.33)	142 per 1.000	**38 fewer per 1.000** (85 fewer to 47 more)
Laboratory-confirmed influenza	3937 (4 RCTs)	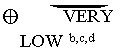	**RR 1.07** (0.83 to 1.39)	69 per 1.000	**5 more per 1.000** (12 fewer to 27 more)

* Outcomes, numbers of events and totals are adjusted accounting for clustering.

**The risk in the intervention group (and its 95% confidence interval) is based on the assumed risk in the comparison group and the **relative effect** of the intervention (and its 95% CI).

**CI:** Confidence interval; **RR:** Risk ratio

**Explanations**

a. baseline imbalance

b. no specific population for COVID-19

c. number of events < 400 or 95% Confidence Interval overlaps threshold for benefit (Guyatt et al. GRADE guidelines 6. Rating the quality of evidence- Imprecision.J Clin Epidemiol. 2011)

d. selection bias (baseline imbalance and unclear allocation)

**GRADE Working Group grades of evidence**

**High certainty:** We are very confident that the true effect lies close to that of the estimate of the effect

**Moderate certainty:** We are moderately confident in the effect estimate: The true effect is likely to be close to the estimate of the effect, but there is a possibility that it is substantially different

**Low certainty:** Our confidence in the effect estimate is limited: The true effect may be substantially different from the estimate of the effect

**Very low certainty:** We have very little confidence in the effect estimate: The true effect is likely to be substantially different from the estimate of effect

### Secondary outcomes

The quality for the evidence was very low for all the secondary outcomes (Table 2). A trend in favour of N95 was found for ILI (4 RCTs, 8220 HCWs; RR 0.72, 95% CI 0.38, 1.37; I^2^ = 24%), laboratory confirmed respiratory viral infections (3 RCTs, 3040 HCWs; RR 0.84, 95% CI 0.52, 1.34; I^2^ = 0%), laboratory confirmed respiratory infection (2 RCTs, 6221 HCWs; RR 0.73, 95% CI 0.40, 1.33; I^2^ = 69%), laboratory confirmed influenza (4 RCTs, 8220 HCWs; RR 1.07, 95% CI 0.83, 1.39; I^2^ = 0%). The protective effect of N95 respirators for bacterial respiratory colonisation was significant (2 RCTs, 2594 HCWs; RR 0.41, 95% CI 0.28, 0.61; I^2^ = 0%).

Discomfort was higher among HCWs wearing N95 respirators [38]: data are reported through descriptive statistics in S1 Appendix. Outcome results—forest plots of adjusted meta-analysis.

## Discussion

The exceptional threat to the resilience of health care systems posed by this pandemic is well known and protecting HCWs is among the most important interventions for successfully managing the COVID-19 pandemic. There is no agreement among healthcare organisations on whether HCWs should wear surgical masks or N95 respirators during the routine care (not involving aerosol generating procedures) of COVID-19 suspected or affected patients [6–8].

While some observational evidence suggests that an airborne (aerosol) route of diffusion of SARS-CoV-2 may occur also outside the aerosol generating procedures (such as tracheal intubation, sputum induction and airway suctioning) so far no RCT has directly compared the effect of filtering respirators with surgical masks in preventing SARS-CoV-2 infection and related deaths among HCWs. Also, it is unlikely that such a trial could be ethically acceptable in the near future if the evidence of an aerosol diffusion of SARS-CoV-2 grows even more. Indeed, in our meta-analysis we found that during an infectious outbreak wearing N95 halves the risk of any clinical respiratory infection in HCWs compared to wearing only surgical masks. In order to support public health decisions regarding the emergency of COVID-19, we introduced the 90% CIs providing a simple extension of standard metanalysis by comparing the potential health benefits of further research and the immediate implementation the findings of existing research. On this basis, an immediate implementation of the intervention (wearing N95 respirator by HCWs) could actually avoid 73 respiratory infections per 1000 HCWs whereas the added value of further research would be to avoid no more than 51 infections per 1000 HCWs, in the worst case scenario, making deferral of this intervention while awaiting more studies unreasonable. The favourable (albeit not significant) trend of N95 for laboratory confirmed respiratory infections and ILI deserves some comment. In fact, these findings could be viewed as evidence against the benefit of respirators as laboratory-confirmed influenza seems to be. Instead, given the blurred distinction between airborne and droplet diffusion of respiratory viruses [42] it could also be considered as indirect evidence of the opportunistic airborne route of transmission of respiratory viruses in the healthcare environment, where prolonged exposures, high viral loads, asymptomatic carriers, overcrowding and poor ventilation could enhance the opportunistic airborne diffusion among HCWs of viruses such as SARS-CoV-2. Regarding the lack of apparent benefit of N95 for influenza in the only trial where this outcome was assessed, both the outpatient setting (with lower viral exposure loads) [43] and the droplet route of transmission believed to be operative for influenza are worth of consideration. Finally, we suggest to integrate the perspective and the findings of this review into the appropriate GRADE framework, considering the added difficulties of urgency and uncertainty, which make the production of a reliable guideline even more challenging [44]. Such guidelines should explicitly consider among other factors the human and organizational costs of delaying the adoption of N95 respirators versus the benefits of an immediate adoption and, finally, the key value of safeguarding HCWs in the context of SARS-CoV-2 pandemic.

### Limitations and strengths

Several limitations should be considered. First, a review protocol of this systematic review was not made publicly available in PROSPERO due to the need to publish timely a review on COVID-19. Second, wearing N95 respirators is only one component among a series of complex procedures, so that the identified effect cannot exclusively be attributed to this intervention. The source of infection (community rather than the workplace) cannot be ascertained in any of the trials. Third, one RCT required HCWs to wear N95 respirators only when caring for patients with febrile respiratory illness [37], whereas all others specified continuous respirator use. While, one study was performed in an outpatient setting that can be considered at moderate risk of transmission [5]. Finally, our meta-analyses did not investigate the adherence of wearing an N95 respirator. One of the included trials reported discomfort of using N95 respirators [38]. As well, we did not anticipate mortality as outcome in the review because of the low mortality rate of SARS-CoV-2 irrespectively of being HCWs or not. Indeed, the mortality rate among HCWs should be related to the infection rate, which was our primary outcome. Thus, we call for future studies investigating the mortality rate in HCWs.

The main strength of our study was the use of appropriate Cochrane methods for analysing cluster randomized studies. By inflating variances this method allows to obtain adjusted estimates of relative risks. Indeed, if clustering is ignored, P values will be artificially small resulting in false positive conclusions about the effectiveness of the intervention. In addition, we adopted the Claxton model to quantify the trade-off between immediate implementation of the intervention versus deferring it while awaiting further evidence. Finally, we offered a solid ground for the development of future evidence-based clinical guidance based on the quality of evidence by GRADE approach which can be applied in a variety of decision-making contexts, including urgent responses as the novel coronavirus SARS-COV-2 pandemic [45, 46].

## Conclusion

This is the first systematic review on the efficacy of N95 respirators versus surgical masks among HCWs accounting for possible bias derived from cluster trials and evaluating the findings from a public health policy perspective. We found evidence that N95 respirators halve the risk of any respiratory infection compared to surgical masks. Considering that the absenteeism from work due to healthcare related infections hampers heavily the resilience of healthcare systems facing an infectious pandemic, the protective effect of N95 respirators for this primary outcome could produce large benefits in the current context. Furthermore, the immediate implementation of the intervention, rather than deferring it until more studies will be available, seems justified on a sound quantitative basis. The evidence from the current study could be used to inform the production of trustworthy GRADE based guidelines for the prevention of SARS-CoV-2 infection among HCWs.

## Supporting information

S1 Checklist PRISMA 2009 checklist. (DOC)

S1 Appendix (DOCX)
