# Supplementary material for: The need of health policy perspective to protect Healthcare Workers during COVID-19 pandemic. A GRADE rapid review on the N95 respirators effectiveness
Source: PLoS One. 2020 Jun 3;15(6):e0234025. doi: 10.1371/journal.pone.0234025 (PMC7269249; doi:10.1371/journal.pone.0234025)
Supplement: S1 Appendix — (DOCX) [file pone.0234025.s002.docx]

**S1 Appendix**

[METHODS 2](#_Toc39588823)

[S1. Outcomes and assessment measurements 2](#_Toc39588824)

[S1. Search strategy 3](#_Toc39588825)

[S1. Risk of bias assessment. 4](#_Toc39588826)

[S1. Data Analysis and Synthesis of Results 4](#_Toc39588827)

[S1. Quality of the evidence-GRADE approach 4](#_Toc39588828)

[RESULTS 5](#_Toc39588829)

[S1. List of excluded studies. 5](#_Toc39588830)

[S1. Results of Risk of bias assessment 6](#_Toc39588831)

[S1. Outcome results - forest plots of adjusted meta-analysis. 7](#_Toc39588832)

[3.1. Clinical Respiratory Illness (CRI) – primary outcome 7](#_Toc39588833)

[3.2. Influenza like illness (ILI) – secondary outcome 8](#_Toc39588834)

[3.3. Laboratory-confirmed respiratory viral infections – secondary outcome 9](#_Toc39588835)

[3.4. Laboratory-confirmed bacterial colonization – secondary outcome 10](#_Toc39588836)

[3.5. Laboratory-confirmed respiratory infection – secondary outcome 10](#_Toc39588837)

[3.6. Laboratory-confirmed influenza – secondary outcome 11](#_Toc39588838)

[3.7 Discomfort of wearing respiratory protections – not secondary outcome 11](#_Toc39588839)

[References 12](#_Toc39588840)

# METHODS

## S1. Outcomes and assessment measurements

We identified a priori the primary and secondary outcomes.

*Primary outcome*:

1. *SARS-CoV-2 infection* defined as patient with acute respiratory tract infection (sudden onset of at least one of the following: cough, fever, shortness of breath) AND with no other aetiology that fully explains the clinical presentation AND with a history of travel or residence in a country/area reporting local or community transmission* during the 14 days prior to symptom onset; or a patient with any acute respiratory illness AND having been in close contact with a confirmed or probable COVID-19 case in the last 14 days prior to onset of symptoms; a patient with severe acute respiratory infection (fever and at least one sign/symptom of respiratory disease (e.g., cough, fever, shortness breath)) AND requiring hospitalisation (SARI) AND with no other aetiology that fully explains the clinical presentation [1].
2. C*linical respiratory illness* *(CRI)* defined as two or more respiratory symptoms or one respiratory symptom and a systemic symptom;

*Secondary outcome*:

1. *Influenza like illness (ILI)* defined as temperature of at least 37.8°C plus at least 1 respiratory symptom (sore throat, cough, sneezing, runny nose, nasal congestion, headache), with or without laboratory confirmation;
2. *Laboratory-confirmed respiratory viral infections* defined as detection of virus such as adenoviruses, human metapneumovirus, coronaviruses 229E/NL63 and OC43/HKU1, parainfluenza viruses 1, 2 and 3, influenza viruses A and B, respiratory syncytial viruses A and B, or rhinovirus A/B by nucleic acid testing (NAT);
3. *Laboratory-confirmed bacterial colonization* defined as detection of Streptococcus pneumoniae, legionella, Bordetella pertussis, chlamydia, Mycoplasma pneumoniae, or Haemophilus influenzae type B by multiplex polymerase chain reaction;
4. *Laboratory-confirmed respiratory infection* defined as detection of a respiratory pathogen by polymerase chain reaction or serological evidence of infection with a respiratory pathogen*;*
5. *Laboratory-confirmed influenza* defined as detection of viral infection such as of adenoviruses; human metapneumovirus, coronaviruses 229E/NL63 and OC43/HKU1, parainfluenza viruses 1, 2, and 3; influenza viruses A and B; respiratory syncytial viruses A and B; or rhinoviruses A/B.

*Not important outcome:*

1. *Discomfort of wearing respiratory protections.*

## S1. Search strategy

PubMed

"Meta-Analysis" [Publication Type] OR "Systematic Review" [Publication Type] OR "meta-analysis" OR "systematic review" OR Randomized Controlled Trial[ptyp] OR Controlled Clinical Trial[ptyp] OR randomized[Title/Abstract] OR randomly [Title/Abstract] OR trial[Title/Abstract] OR group[Title/Abstract]) AND ("Respiratory Protective Devices"[Mesh] OR "Masks"[Mesh] OR mask* OR facemask* OR N95* OR N-95*) AND ("Severe Acute Respiratory Syndrome"[Mesh] OR "Coronavirus"[Mesh] OR "Influenza, Human"[Mesh] OR flu OR influenza OR grippe OR SARS OR “severe acute respiratory syndrome”))

Embase

('meta analysis'/exp OR 'meta analysis':ab,ti OR 'systematic review'/exp OR 'systematic review':ab,ti OR 'randomized controlled trial'/exp OR 'randomized':ab,ti) AND ('surgical mask'/exp OR 'gas mask'/exp OR mask* OR facemask* OR 'n95 respirator') AND ('severe acute respiratory syndrome'/exp OR 'influenza'/exp OR flu OR influenza OR sars OR 'severe acute respiratory syndrome') AND [embase]/lim

The Cochrane Library

#1 MeSH descriptor: [Influenza, Human] explode all trees

#2 MeSH descriptor: [Severe Acute Respiratory Syndrome] explode all trees

#3 flu OR influenza OR SARS OR 'severe acute respiratory syndrome'

#4 MeSH descriptor: [Masks] explode all trees

#5 mask* OR facemask* OR 'N95' or 'N-95'

#6 #1 or #2 or #3

#7 #4 or #5

#8 #6 AND #7 in Trials

## S1. Risk of bias assessment

Two reviewers independently assessed the risk of bias of the selected RCTs using the Cochrane Risk of Bias tool, which includes domains on random sequence generation, allocation concealment, blinding of participants and personnel, blinding of outcome assessors, incomplete outcome data, and selective reporting. Thus, in cluster-randomized trials, particular biases were considered: (i) recruitment bias; (ii) baseline imbalance; (iii) loss of clusters; (iv) incorrect analysis; and (v) comparability with individually randomized trials [2]. Each item has been scored as “high,” “low,” or “unclear” RoB if no sufficient information is reported. Two reviewers independently assessed the above-mentioned domains of RoB. Any disagreement was resolved by consensus.

## S1. Data Analysis and Synthesis of Results

The table below reports the intraclass correlation coefficient (ICC) for each included study, when available.

| **ID** | **RCTs** | **Cluster** | **Setting** | **ICC** | **Design Effect** |
| --- | --- | --- | --- | --- | --- |
| 1 | Loeb 2009 | No | Hospital | - | - |
| 3 | MacIntyre 2011 | Yes | Hospital | 0.01 | 2 |
| 4 | MacIntyre 2013 | Yes | Hospital | 0.027 | 1.65 |
|  | MacIntyre 2014* | Yes | Hospital | 0.01 | 2 |
| 5 | Radonovich 2019 | Yes | Outpatients | 0.1 | 2.5 |

Legend:*This publication is part of study of MacIntyre 2011 showing different outcomes.

## S1. Quality of the evidence-GRADE approach

We evaluated the overall quality of the evidence for primary and secondary outcomes using the Grading of Recommendations Assessment, Development and Evaluation (GRADE) [3]. Five GRADE domains - study limitations, consistency of effect, imprecision, indirectness and publication bias – were analytically assessed. The final judgement on certainty was revised and downgraded eventually, by one or two levels, reflecting the extent of bias in important quality domains. Adjusted estimates were considered for judging the imprecision domain. For primary outcomes, absolute effects were calculated at 95% CI and 90% CI[4]. A ‘summary of findings’ including the quality of the evidence, reasons for limitation and main findings were displayed in table. We used the GRADEpro software [5] to present findings.

# RESULTS

## S1. List of excluded studies.

| 1 | Atrie, D. & Worster, A. Surgical mask versus N95 respirator for preventing influenza among health care workers: a randomized trial. Cjem 14, 50-52. | Journal club (referred to Loeb 2009 included) |
| --- | --- | --- |
| 2 | Bin-Reza, F., Lopez Chavarrias, V., Nicoll, A., Chamberl & , M.E. The use of masks and respirators to prevent transmission of influenza: a systematic review of the scientific evidence. Influenza Other Respir Viruses 6, 257-267 wrong comparisons: studies already included | Wrong comparison – studies already included |
| 3 | Canini, L., Andréoletti, L., Ferrari, P., et al. Surgical masks nearly as effective as N95 respirators, study finds Surgical mask to prevent influenza transmission in households: a cluster randomized trial. | Wrong comparisons: surgical mask versus no intervention |
| 4 | Cowling, B.J., Zhou, Y., Ip, D.K., Leung, G.M. & Aiello, A.E. 2010 Face masks to prevent transmission of influenza virus: a systematic review. Epidemiol Infect 138, 449-456. | Wrong comparison – studies already included |
| 5 | Results of the Respiratory Protection Effectiveness Clinical Trial (ResPECT): Radonovich, 2018 | Duplicate – additional reference of included study |
| 6 | Jefferson, T., Del Mar, C., Dooley, L., et al. Physical interventions to interrupt or reduce the spread of respiratory viruses: systematic review. Bmj 339, b3675. | Wrong comparison – studies already included |
| 7 | Jefferson, T., Foxlee, R., Del Mar, C., et al. 2011 Interventions for the interruption or reduction of the spread of respiratory viruses. Cochrane Database Syst Rev, Cd006207. | Wrong comparison – studies already included |
| 8 | Jefferson, T., Foxlee, R., Del Mar, C., et al. Physical interventions to interrupt or reduce the spread of respiratory viruses: systematic review. Bmj 336, 77-80. | Wrong comparison – studies already included |
| 9 | Long, Y., Hu, T., Liu, L., et al. Effectiveness of N95 respirators versus surgical masks against influenza: A systematic review and meta-analysis. J Evid Based Med. | Wrong comparison – studies already included |
| 10 | MacIntyre, C.R., Cauchemez, S., Dwyer, D.E., et al. 2009 Face mask use and control of respiratory virus transmission in households. Emerg Infect Dis 15, 233-241. | Wrong population |
| 11 | MacIntyre, C.R., Chughtai, A.A., Rahman, B., et al. 2017 The efficacy of medical masks and respirators against respiratory infection in healthcare workers. Influenza Other Respir Viruses 11, 511-517. | This study is a pooled analysis of two trials. |
| 12 | Offeddu, V., Yung, C.F., Low, M.S.F. & Tam, C.C. Effectiveness of Masks and Respirators Against Respiratory Infections in Healthcare Workers: A Systematic Review and Meta-Analysis. Clin Infect Dis 65, 1934-1942. wrong comparisons: studies already included | Wrong comparison – studies already included |
| 13 | Radonovich, L.J., Jr., Bessesen, M.T., Cummings, D.A., et al. 2016 The Respiratory Protection Effectiveness Clinical Trial (ResPECT): a cluster-randomized comparison of respirator and medical mask effectiveness against respiratory infections in healthcare personnel. BMC Infect Dis 16, 243. | Protocol – additional reference of included study |
| 14 | Saunders-Hastings, P., Crispo, J.A.G., Sikora, L. & Krewski, D. Effectiveness of personal protective measures in reducing pandemic influenza transmission: A systematic review and meta-analysis. Epidemics 20, 1-20. wrong comparisons | Wrong comparison – studies already included |
| 15 | Smith, J.D., MacDougall, C.C., Johnstone, J., Copes, R.A., Schwartz, B. & Garber, G.E. Effectiveness of N95 respirators versus surgical masks in protecting health care workers from acute respiratory infection: a systematic review and meta-analysis. Cmaj 188, 567-574. | Wrong comparison – studies already included |
| 16 | Wong, V.W., Cowling, B.J. & Aiello, A.E. Hand hygiene and risk of influenza virus infections in the community: a systematic review and meta-analysis. Epidemiol Infect 142, 922-932. | Wrong comparison |
| 17 | Xiao, J., Shiu, E.Y.C., Gao, H., et al. Nonpharmaceutical Measures for Pandemic Influenza in Nonhealthcare Settings-Personal Protective and Environmental Measures. Emerg Infect Dis 26. | Wrong comparison |

## S1. Results of Risk of bias assessment

Figure 2 shows the risk of bias of included studies: Loeb et al. 2009 [6] showed a low risk of bias as well the other three studies [7-10] were assessed for additional bias related to clustering. Overall, four cluster randomized controlled trial were assessed as high risk of bias for imbalance at baseline (**Figure 3).**


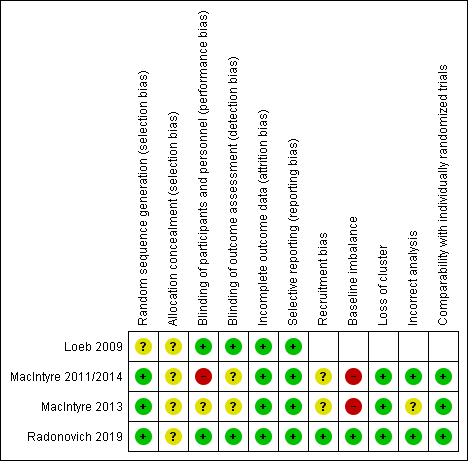


**Figure 3.** Risk of bias summary. *Loeb 2009: risk of bias was assessed as randomized by individual controlled trial. MacIntyre 2011/2014, MacIntyre 2013 and Radonovich 2019: risk of bias was assessed as randomized cluster controlled trial.

## S1. Outcome results - forest plots of adjusted meta-analysis.

For each outcome, both fixed and random effects model meta-analysis are reported.

### **3.1. Clinical Respiratory Illness (CRI) – primary outcome**

We included two cluster RCTs in inpatients hospital setting [7, 9]. Adjusting data for clustering, using N95 respirators statistically significantly reduced the risk of developing a clinical respiratory illness versus the use of the surgical mask, the estimate did not change assuming either a fixed effects model or a random effects model (RR 0.43, 95% CI 0.29, 0.64; I^2^=0%).


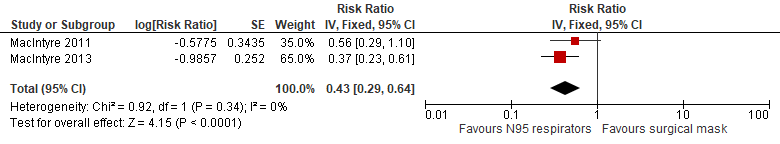

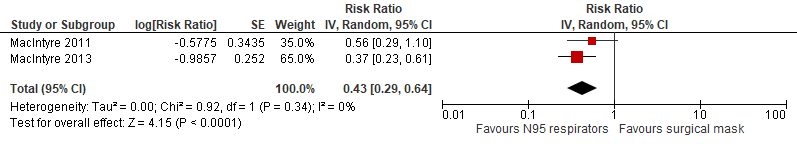


### **3.2. Influenza like illness (ILI) – secondary outcome**

Four studies reported useful data for the analysis. We included one randomized controlled trial [6] and four cluster randomized trials [7, 9, 10]. The use of N95 respirator seems to not reduce the risk of developing an ILI than wearing the surgical mask: the effect was not significant assuming either a fixed effects model (RR 0.78, 95% CI 0.55, 1.10; I^2^=24%) or a random effects model (RR 0.72, 95% CI 0.38, 1.37; I^2^=24%).


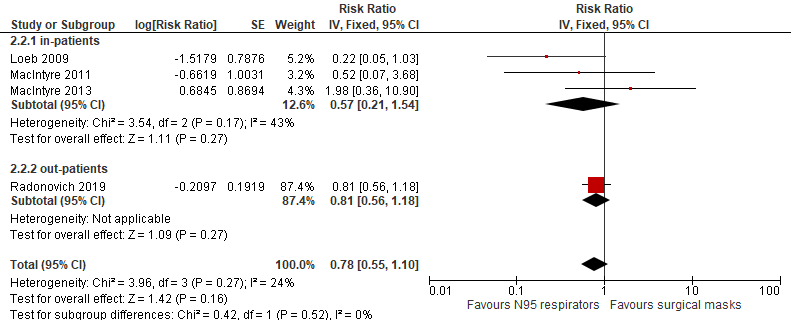


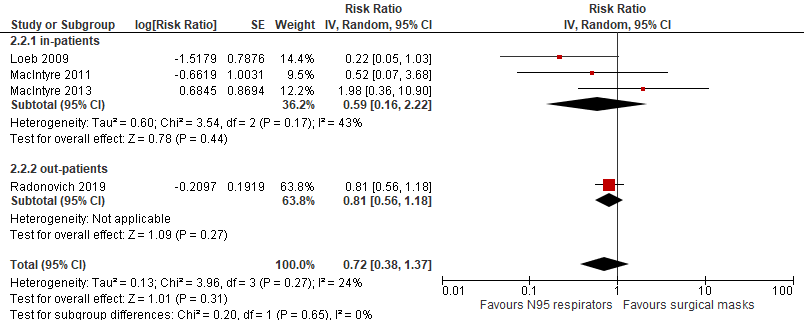


### **3.3. Laboratory-confirmed respiratory viral infections – secondary outcome**

Three studies reported useful data for the analysis. We included one randomized controlled trial [6] and two cluster randomized trials [7, 9]. Adjusting data for clustering, using N95 respirator reduced the risk of having a laboratory confirmed respiratory viral infection than using the surgical mask but, the effect is not statistically significant and the estimate did not change assuming either a fixed effects model or a random effects model (RR 0.84, 95% CI 0.52, 1.34; I^2^=0%).


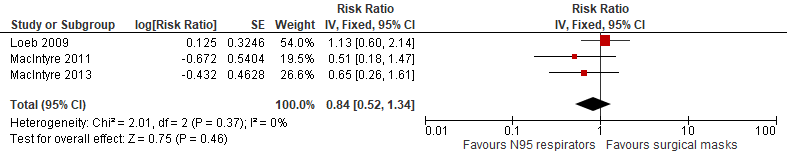


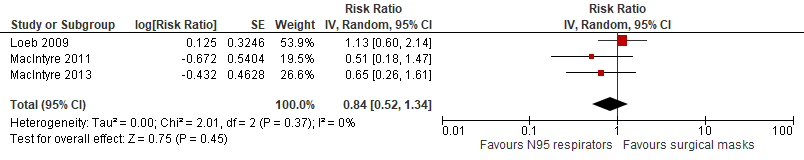


### **3.4. Laboratory-confirmed bacterial colonization – secondary outcome**

Two cluster RCTs reported useful data for the meta-analysis [7, 8]. Adjusting data for clustering, using N95 respirator reduced the risk of having a laboratory confirmed bacterial colonization than using the surgical mask with a statistically significant effect and the estimate did not change assuming either a fixed effects model or a random effects model (RR 0.41, 95% CI 0.28, 0.61; I^2^=0%).


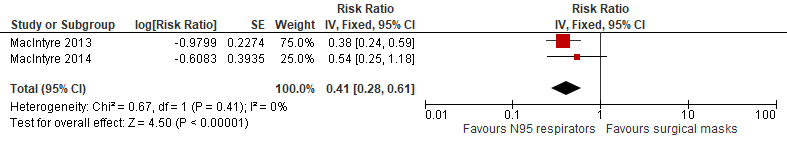


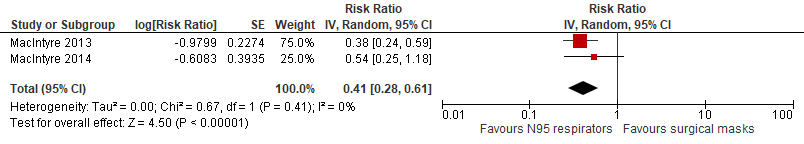


### **3.5. Laboratory-confirmed respiratory infection – secondary outcome**

Two cluster RCTs reported useful data for the meta-analysis [8, 10]. Studies were performed in a hospital inpatients and outpatients setting. Adjusting data for clustering, using N95 respirator reduced the risk of having a laboratory confirmed respiratory infection than using the surgical mask, but the effect is not statistically significant assuming either a fixed effects model (RR 0.86, 95% CI 0.69, 1.08; I^2^=69%) or a random effects model (RR 0.73, 95% CI 0.40, 1.33; I^2^=69%).


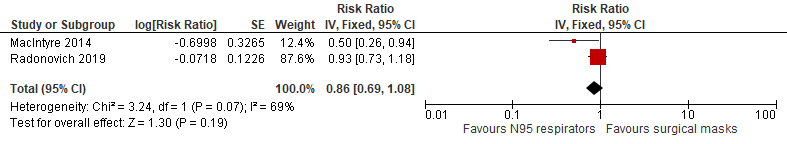

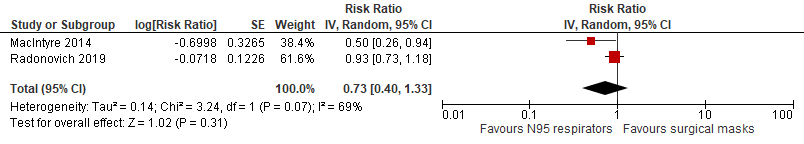


### **3.6. Laboratory-confirmed influenza – secondary outcome**

Four studies reported useful data for the analysis. We included one randomized controlled trial [6] and four cluster randomized trials [7, 9, 10]. Adjusting data for clustering, the use of N95 respirator did not statistically significantly reduced the risk of detecting a laboratory-confirmed influenza than wearing the surgical mask assuming either a fixed or a random effects model (RR 1.07, 95% CI 0.83, 1.39; I^2^=0%).


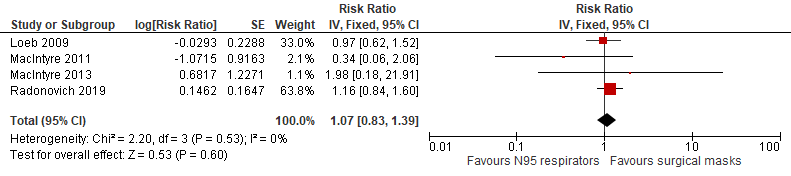

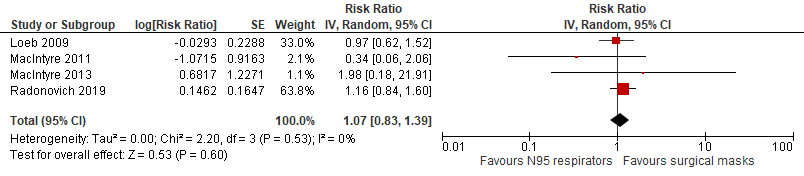


### **3.7 Discomfort of wearing respiratory protections – not secondary outcome**

Only 1 cluster RCT collected data on discomfort of wearing N95 respirators and surgical masks reported by health care workers [9]. In surgical mask group, the 85.5% (out of 492 HCW) reported no problems associated with the using of masks but a 9∙8% stated that was uncomfortable; whereas, difficulty breathing, pressure on nose and allergies were the three most reported discomfort wearing the mask (respectively, 12.5%(35/281); 11%(31/281); 9.3%(26/281)). On the contrary, in the N95 respirators group the 47.7% (out of 949 HCW) reported no problems associated with the using of respirator but 41.9% stated that was uncomfortable; whereas, pressure on nose, difficulty breathing, and headaches were the three most reported discomfort wearing the respirator (respectively, 52.2%(366/701); 19.4% (136/701); 13.4% (94/701)).

# References

1. World Health Organisation (WHO), 2020. Novel Coronavirus (2019-nCoV).

2. Higgins JPT GS. Cochrane Handbook for Systematic Reviews of Interventions. Chapter 16: Special topics in statistics. Version 5.1.0 [updated March 2011]. The Cochrane Collaboration, 2011. [Available from: <https://handbook-5-1.cochrane.org/chapter_16/16_3_2_assessing_risk_of_bias_in_cluster_randomized_trials.htm>.

3. Guyatt G, Oxman AD, Akl EA, Kunz R, Vist G, Brozek J, et al. GRADE guidelines: 1. Introduction-GRADE evidence profiles and summary of findings tables. J Clin Epidemiol. 2011;64(4):383-94.

4. Guyatt GH, Oxman AD, Santesso N, Helfand M, Vist G, Kunz R, et al. GRADE guidelines: 12. Preparing summary of findings tables-binary outcomes. J Clin Epidemiol. 2013;66(2):158-72.

5. GRADEpro Guideline Development Tool. GRADEpro GDT. McMaster University (Developed by Evidence Prime, Inc.) (2015). Available from: <https://gradepro.org/cite.html> (Accessed: March 07, 2019).

6. Loeb M, Dafoe N, Mahony J, John M, Sarabia A, Glavin V, et al. Surgical mask vs N95 respirator for preventing influenza among health care workers: a randomized trial. Jama. 2009;302(17):1865-71.

7. MacIntyre CR, Wang Q, Seale H, Yang P, Shi W, Gao Z, et al. A randomized clinical trial of three options for N95 respirators and medical masks in health workers. Am J Respir Crit Care Med. 2013;187(9):960-6.

8. MacIntyre CR, Wang Q, Rahman B, Seale H, Ridda I, Gao Z, et al. Efficacy of face masks and respirators in preventing upper respiratory tract bacterial colonization and co-infection in hospital healthcare workers. Prev Med. 2014;62:1-7.

9. MacIntyre CR, Wang Q, Cauchemez S, Seale H, Dwyer DE, Yang P, et al. A cluster randomized clinical trial comparing fit-tested and non-fit-tested N95 respirators to medical masks to prevent respiratory virus infection in health care workers. Influenza Other Respir Viruses. 2011;5(3):170-9.

10. Radonovich LJ, Jr., Simberkoff MS, Bessesen MT, Brown AC, Cummings DAT, Gaydos CA, et al. N95 Respirators vs Medical Masks for Preventing Influenza Among Health Care Personnel: A Randomized Clinical Trial. Jama. 2019;322(9):824-33.
